# Multi-Agent Reinforcement Learning via Adaptive Kalman Temporal Difference and Successor Representation

**DOI:** 10.3390/s22041393

**Published:** 2022-02-11

**Authors:** Mohammad Salimibeni, Arash Mohammadi, Parvin Malekzadeh, Konstantinos N. Plataniotis

**Affiliations:** 1 Concordia Institute for Information System Engineering, Concordia University, Montreal, QC H3G 1M8, Canada; m_alimib@encs.concordia.ca; 2 Department of Electrical and Computer Engineering, University of Toronto, Toronto, ON M5S 3G8, Canada; p_malekz@encs.concordia.ca (P.M.); kostas@ece.utoronto.ca (K.N.P.)

**Keywords:** Kalman Temporal Difference, Multiple Model Adaptive Estimation, Multi-Agent Reinforcement Learning, Successor Representation

## Abstract

Development of distributed Multi-Agent Reinforcement Learning (MARL) algorithms has attracted an increasing surge of interest lately. Generally speaking, conventional Model-Based (MB) or Model-Free (MF) RL algorithms are not directly applicable to the MARL problems due to utilization of a fixed reward model for learning the underlying value function. While Deep Neural Network (DNN)-based solutions perform well, they are still prone to overfitting, high sensitivity to parameter selection, and sample inefficiency. In this paper, an adaptive Kalman Filter (KF)-based framework is introduced as an efficient alternative to address the aforementioned problems by capitalizing on unique characteristics of KF such as uncertainty modeling and online second order learning. More specifically, the paper proposes the Multi-Agent Adaptive Kalman Temporal Difference (MAK-TD) framework and its Successor Representation-based variant, referred to as the MAK-SR. The proposed MAK-TD/SR frameworks consider the continuous nature of the action-space that is associated with high dimensional multi-agent environments and exploit Kalman Temporal Difference (KTD) to address the parameter uncertainty. The proposed MAK-TD/SR frameworks are evaluated via several experiments, which are implemented through the OpenAI Gym MARL benchmarks. In these experiments, different number of agents in cooperative, competitive, and mixed (cooperative-competitive) scenarios are utilized. The experimental results illustrate superior performance of the proposed MAK-TD/SR frameworks compared to their state-of-the-art counterparts.

## 1. Introduction

Reinforcement Learning (RL), as a class of Machine Learning (ML) techniques, targets providing human-level adaptive behavior by construction of an optimal control policy [1]. Generally speaking, the main underlying objective is learning (via trial and error) from previous interactions of an autonomous agent and its surrounding environment. The optimal control (action) policy can be obtained via RL algorithms through the feedback that environment provides to the agent after each of its actions [2,3,4,5,6,7,8,9]. Policy optimality can be reached via such an approach with the goal of increasing the reward over time. In most of the successful RL applications, e.g., Go and Poker games, robotics, and autonomous driving, typically, several autonomous agents are involved. This naturally falls within the context of Multi-Agent RL (MARL), which is a relatively long-established domain; however, it has recently been revitalized due to the advancements made in the single-agent RL approaches. In the MARL domain, which is the focus of this manuscript, multiple decision-making agents interact (cooperate and/or compete) in a shared environment to gain a common or a conflicting goal. Research Questions: In this paper, we aim to answer the following research questions:

- How to tackle overfitting, high sensitivity to parameter selection, and sample inefficiency issues of MARL, typically, associated with DNN-based solutions?
- How to properly handle a change in the reward model for learning the underlying value function and how to capture uncertainty of the Successor Representation (SR)?
- How multi-agent adaptive Kalman Temporal Difference (KTD) can be adopted to work within the SR formulation?
- Ho to find a trade-off between exploration and exploitation of MARL?

Challenges: To address the aforementioned research questions, we faced the following challenges:

- Learning localized reward functions and dealing with the lack of prior knowledge on observation noise covariance and observation mapping function.
- Selecting KF parameters for learning the reward function as its performance is highly dependent on these values.
- Encoding continuous states into feature vectors and projecting the reward function as a linear function of the extracted features.
- Adopting KTD approach to the SR learning procedure.
- Capturing the uncertainty associated with the SR and calculating the value function based on the learned SR values and the reward function.
- Exploration/exploitation trade-off, i.e., to select from actions with known associated rewards or explore new possible actions with unknown rewards.

Before, introducing contributions of the paper and its novelties, first, a brief literature review is provided next.

Literature Review: Traditionally, RL algorithms are classified as (i) Model-Free (MF) approaches [4,10,11] where sample trajectories are exploited for learning the value function, and (ii) Model-Based (MB) techniques [12] where reward functions are estimated by leveraging search trees or dynamic programming [13]. MF methods, generally, do not adapt quickly to local changes in the reward function. On the other hand, MB techniques can adapt quickly to changes in the environment, but this comes with a high computational cost [14,15,16]. To address the above adaptation problems, Successor Representation (SR) approaches [17,18] are proposed as an alternative RL category. The SR method provides the flexibility of the MB algorithm and has computational efficiency comparable to that of the MF algorithms. In SR-based methods, both the immediate reward expected to be received after each action and the discounted expected future state occupancy (which is called the SR) are learned. Afterwards, in each of the successor states, the value function is factorized into the SR and the immediate reward. This factorization only needs learning of the reward function for new tasks, allowing rapid policy evaluation when reward conditions are changed. In scenarios with a limited number of states, the SR and the reward function (thus, the value function) associated with each state can be readily computed. Computation of the value function, however, is infeasible for MARL problems, as in such scenarios we deal with a large number of continuous states [19]. In other words, conventional approaches developed for single agent scenarios such as single-agent SR, Q-Learning, or policy gradient cannot be directly adopted to MARL to compute the value function. The main problem here is that, typically, from a single agent’s perspective, the environment tends to become unstable as each agent’s policies change during the training process. In the context of deep Q-learning [20], this leads to stabilization issues as it is difficult to properly use the previous localized experiences. From the perspective of policy gradient, typically, observations demonstrate high variance in coordinating multiple agents.

To leverage SR-based solutions for MARL, value function approximation is unavoidable, and one can use either linear or non-linear estimation approaches [21,22]. In both categories, a set of adjustable parameters define the value of the approximated function. Non-linear function approximators, such as Deep Neural Networks (DNNs) [21,23,24,25], have enabled application of RL methods to complex multi-agent scenarios. While DNN approaches like Deep Q-Networks (DQN) [26] and Deep Deterministic Policy Gradient (DDPG) [27] achieved superior results, they suffer from some major disadvantages including the overfitting problem, high sensitivity in choosing parameters, sample inefficiency, and high number of episodes required for training the models. The linear function approximators, on the other hand, transform the approximation problem into a weight calculation problem in order to fuse several local estimators. Convergence can be examined when linear function approximators are utilized, as they are better understood than their non-linear counterparts [28,29]. Cerebellar Model Articulation Controllers (CMACs) [30] and Radial Basis Functions (RBFs) [31] are usually used as linear estimators in this context. It has been shown, however, that the function approximation process can be better represented via gradual-continuous transitions [32]. Albeit the computation of the RBFs’ parameters is usually based on prior knowledge of the problem at hand, these parameters can also be adapted leveraging observed transitions in order to improve the autonomy of the approach. In this context, cross entropy and gradient descent methods [33] can be utilized for the adaptation task. Stability of the gradient descent-based approach was later improved by exploiting a restrictive method in [32], which is adopted in this manuscript.

After verifying the value function’s structure, to train the value function approximator, the following methodologies can be used: (i) Bootstrapping methods, e.g., Fixed-Point Kalman Filter (FPKF) [34]; (ii) Residual techniques such as Kalman Temporal Difference (KTD) and Gaussian Process Temporal Difference (GPTD) [35], which is a special form of the KTD; and (iii) Projected fixed-point methods such as Least Square Temporal Difference (LSTD) [36]. Among these methodologies, KTD [37] is a prominent technique as, based on the selected structure, it provides both uncertainty and Minimum Mean Square Error (MMSE) approximation of the value function. In particular, uncertainty is beneficial for achieving higher sample efficiency. The KTD approach, however, requires prior knowledge of the filter’s parameters (e.g., noise covariance of the process and measurement models), which are not readily available in realistic circumstances. Parameter estimation is a well-studied problem within the context of Kalman Filtering (KF), where several adaptive schemes are developed over the years including but not limited to Multiple Model Adaptive Estimation (MMAE) methods [38,39,40] and, innovation-based adaptive schemes [41]. When the system’s mode is changing, the latter has the superiority to adapt faster and its efficiency was shown in [42], where different suggested averaging and weighting patterns were compared. MMAE methods were already utilized in the RL problems, for instance, Reference [43] proposed a multiple model KTD coupled with a model selection mechanism to address issues related to the parameter uncertainty. Existing multiple model methodologies are, however, not easily generalizable to the MARL problem.

In methods proposed in [16,44,45,46], while the classical TD learning is coupled with DNNs, uncertainty of the value function and that of the SR is not studied. To deal with uncertainty, a good combination of exploitation and exploration should be used to prevent the agent’s overconfidence about its knowledge to fully rely on exploitation. Alternatively, an agent can perform exploration over other possible actions, which might lead to improved results and a reduction in the uncertainty. Although, from computation points of view, it is intractable to find an optimal trade-off between exploitation and exploration, it has been represented that exploration can benefit from the uncertainty in two separate ways, i.e., through added randomness to the value function, and via shifting towards uncertain action selection [1]. Consequently, the approximated value function’s uncertainty, is a beneficial information for resolving the available conflict between exploration and exploitation [1,47]. It was shown in [47] that the sensitivity of the framework to the parameters of the model can be diminished via uncertainty incorporation within the KTD method. Therefore, the required time and memory to find/learn the best model will be reduced compared to DNN-based methods [16,44,45,46]. The reduced sensitivity in setting the parameters enhances the reproducibility feature of a reliable approach, which leads to regeneration of more consistent outputs while running multiple learning epochs. Consequently, the risk of getting unacceptable results in real scenarios will decrease [48]. Geerts et al. [18] leveraged the KTD framework to estimate the SR for problems with discrete state-spaces, however information related to uncertainty of the estimated SR is not considered in the action selection procedure. We have started our research on signal processing-based RL solutions by introducing the MM-KTD [4,5], which is a multiple model Kalman temporal difference approach for single-agent environments with continuous state-space. The AKF-SR is then proposed in [49], which is an adaptive KF-based successor representation approach developed for single-agent scenarios. This paper targets extending our previous works to multi-agent scenarios with heterogenous and continuous state-spaces.

Contributions: The paper proposes a Multi-Agent Adaptive Kalman Temporal Difference (MAK-TD) framework and its SR-based variant, the Multi-Agent Adaptive Kalman Successor Representation (MAK-SR) framework. MAK-TD/SR frameworks consider the continuous nature of the action-space that is associated with high dimensional multi-agent environments and exploit KTD to address the parameter uncertainty. By leveraging the KTD framework, SR learning procedure is modeled into a filtering problem in this work. Intuitively speaking, the goal is to take advantage of the inherent benefits of the KF, i.e., online second-order learning, uncertainty estimation, and non-stationary handling. Afterwards, RBF-based estimation is utilized within the MAK-TD/SR frameworks in order for continuous states to be encoded into feature vectors and for the reward function to be projected as a linear function of the extracted feature vectors. On the other hand, for learning localized reward functions, we resort to MMAE as a remedy to deal with the lack of prior knowledge on observation noise covariance and observation mapping function. Targeting the identified research questions and by addressing the aforementioned challenges, in summary, the paper makes the following key contributions:

- Within the MARL domain, the so-called MAK-TD framework is proposed as compensation for the information inadequacy about a key unknown filter’s parameter, which is the measurement noise covariance. For learning the optimal policy and to simultaneously enhance sample efficiency of the proposed MAK-TD, an off-policy Q-learning approach is implemented.
- MAK-TD is extended to MAK-SR by incorporation of the SR learning process into the filtering problem using KTD formulation for learned SR uncertainty approximation. Moreover, adopting KTD is beneficial to reduce the required memory/time to learn the SR while reducing the model’s sensitivity to parameters selection (i.e., more reliability) in comparison to DNN-based algorithms.
- A coupled gradient descent and MMAE-based approach is adopted for development of the MAK-SR framework to form a KF-based approximation of the reward function. Via the utilized MMAE formulation, sensitivity to prior knowledge on KF key parameters is reduced.
- For establishing a trade-off between exploration and exploitation, an innovative active learning mechanism is implemented to incorporate the uncertainty of the value function obtained from SR learning. Such a mechanism results in efficiently enhancing performance in terms of cumulative reward.

Novelty: The novelty of the proposed frameworks lies in the integration of Kalman temporal different, multiple-model adaptive estimation, and successor representation for MARL problems. Through such an integration, issues related to overfitting and high sensitivity to parameter selection are addressed and changes in the reward model can be accommodated. Furthermore, for establishing a trade-off between exploration and exploitation, an innovative active learning mechanism is implemented to use the obtained uncertainty of the value function. Such a mechanism results in efficiently enhancing performance in terms of cumulative reward.

A multi-agent extension of the OpenAI gym benchmark, a two-dimensional world with continuous space [50] is utilized to simulate cooperative, competitive scenarios, and mix interaction settings. The proposed MAK-TD/SR frameworks are evaluated through a comprehensive set of experiments and simulations illustrating their superior performance compared to their counterparts. The remainder of the paper is organized as follows: In Section 2, the basics of RL and MARL are briefly discussed. The proposed MAK-TD framework is presented in Section 3, and its SR-based variant, the MAK-SR framework, is introduced in Section 4. Experimental results based on multi-agent RL benchmark are presented in Section 5. Section 7, finally, concludes the paper.

## 2. Problem Formulation

To provide the background required for development of the proposed MAK-TD/SR frameworks, in this section, we present an overview of single agent and MARL techniques.

### 2.1. Single-Agent Reinforcement Learning (RL)

In conventional RL scenarios, typically, a single agent is placed in an unknown environment performing autonomous actions with the goal of maximizing its accumulated reward. In such scenarios, the agent starts its interactions with the environment in an initial state denoted by $s_{0}$ and continues to interact with the environment until reaching a pre-defined terminal state $s_{T}$. Action set A is defined from which the agent can select potential actions following a constructed optimal policy. In other words, given its current state $s_{k}\in S$, the single agent follows a policy denoted by $\pi_{k}$ and performs action $a_{k}\in A$ at time *k*. Following the agent’s action, based on transition probability of $P\left(s_{k+1}|s_{k},a_{k}\right)\in P_{a}$, it moves to a new state $s_{k+1}\in S$ receiving reward of $r_{k}\in R$. A discount factor γ∈(0,1) is utilized to incorporate future rewards as such balancing the immediate rewards and future ones. In summary, a Markov Decision Process (MDP), denoted by 5-tuple $\left\{S,A,P_{a},R,\gamma \right\}$, is typically used as the underlying mathematical model that governs the RL process. Therefore, the main objective is learning an optimal policy to map states into actions by maximizing the expected sum of discounted rewards, which is referred to as the optimal policy $\pi^{*}$ [1]. The optimal policy $\pi^{*}$ is typically obtained based on the following state-action value function:

(1) $$\begin{array}{c} Q_{\pi }\left(s,a\right)=E\left\{\sum_{k=0}^{T}\gamma^{k}r_{k}|s_{0}=s,a_{0}=a,a_{k}=\pi \left(s_{k}\right)\right\}. \end{array}$$

Note that in Equation (1), E{·} denotes the expectation operator. To perform an action at the learning stage, the current policy is utilized. Once convergence is reached, $a_{k}=\arg\max_{a\in A}Q_{\pi^{*}}\left(s_{k},a\right)$, which is the optimal policy, can be used by the agent to perform the required tasks. This completes a brief introduction to RL, next, the TD learning is reviewed as a building block of the proposed MAK-TD/SR frameworks.

### 2.2. Off-Policy Temporal Difference (TD) Learning

By taking an action and moving from one state to another, based on the Bellman equation and Bellman update scheme [51], the value function is gradually updated using sample transitions. This procedure is referred to as Temporal Difference (TD) update [51]. There are two approaches to update policy: “on-policy learning” or “off-policy learning”. The former techniques use the current policy for action selection. For example, SARSA [52,53] is an on-policy approach that optimizes the network as

(2) $$\begin{array}{c} Q_{\pi }\left(s_{k},a_{k}\right)=Q_{\pi }\left(s_{k},a_{k}\right)+\alpha (r_{k}+\gamma \;Q_{\pi }\left(s_{k+1},a_{k+1}\right)-Q_{\pi }\left(s_{k},a_{k})\right), \end{array}$$

where α denotes the learning rate and $Q_{\pi }\left(s_{k},a_{k}\right)$ is the state-action value function. In on-policy methods, by following a defined policy, selecting a new state becomes a non-optimal task. Additionally, this approach seems to be inefficient in sample selection since the value function is updated through the current policy instead of using the optimized one. In “off-policy” solutions, such as Q-learning [53,54,55,56], the information received from previous policies is exploited to update the policy and reach a new one (exploitation). On the other hand, to properly explore new states, a stochastic policy is usually chosen as the behavior policy (exploration). In brief, Q-learning is formed based on the Bellman optimal equation as follows:

(3) $$\begin{array}{c} Q_{\pi^{*}}\left(s_{k},a_{k}\right)=Q_{\pi^{*}}\left(s_{k},a_{k}\right)+\alpha \left(r_{k}+\gamma \max_{a\in A}Q_{\pi^{*}}\left(s_{k+1},a\right)-Q_{\pi^{*}}\left(s_{k},a_{k}\right)\right), \end{array}$$

where the optimal policy $\pi^{*}$ is used to form the state-action value function $Q_{\pi^{*}}\left(s_{k},a_{k}\right)$. The policy can be obtained via a greedy approach as follows:

(4) $$\begin{array}{c} V_{\pi^{*}}\left(s\right)=\max_{a\in A}Q_{\pi^{*}}\left(s_{k},a\right). \end{array}$$

Upon convergence, actions can be selected based on the optimal policy and not the behavior policy as follows:

(5) $$\begin{array}{c} a_{k}=\arg\max_{a\in A}Q_{\pi^{*}}\left(s_{k},a\right). \end{array}$$

This completes our discussion on TD learning. In what follows, we discuss the MARL approaches as well as value function approximation using the proposed algorithms in the multi-agent environments.

### 2.3. Multi-Agent Setting

Within the context of MARL, we consider a scenario with *N* agents, each with its localized observations, actions, and states. In other words, Agent *i*, for (1≤i≤N), utilizes policy $\pi^{\left(i\right)}$, which is a function from the Cartesian product of its localized action set $A^{\left(i\right)}$ and its localized observation set $Z^{\left(i\right)}$ to a real number within zero and one. We use superset $S=\{S^{\left(1\right)},\ldots ,S^{\left(N\right)}\}$ to collectively represent all the localized states, $S^{\left(i\right)}$, for (1≤i≤N). Likewise, supersets $A=\{A^{\left(1\right)},\ldots ,A^{\left(N\right)}\}$ and $Z=\{Z^{\left(1\right)},\ldots ,Z^{\left(N\right)}\}$ are used to jointly represent all the localized actions and local observations, respectively. Each agent makes localized decisions following the transition function $T:S\times A^{\left(1\right)}\times ,\ldots ,\times A^{\left(N\right)}\to S^{2}$. Consequently, an action is performed locally resulting in a new localized measurement and a localized reward $r^{\left(i\right)}:S\times A^{\left(i\right)}\to R$. The main objective of each agent is to maximize its localized expected return $R^{\left(i\right)}=\sum_{t=0}^{T}\gamma^{t}{\left(r^{\left(i\right)}\right)}^{t}$ over a termination window of *T* using a predefined discount factor of γ.

Traditional models like policy gradient or Q-Learning are not suitable for MARL scenarios [57], since the policy of an agent changes during the progress of the training, and the environment becomes non-stationary towards that specific agent’s points of view. Consequently, most recently proposed platforms for multi-agent scenarios employ other strategies, where the agents’ own observation (known as local information at the execution time) are exploited to learn optimal localized policies. Typically, such methods do not consider specific communication patterns between agents or any differentiable model of the environment’s dynamics [57]. Moreover, these models support different interactions between agents from cooperation to competition or their combination [57,58]. In this context, an adaptation is made between the decentralized execution and centralized training to be able to feed the policy training steps with more available data to speed up the process of finding the optimal policy.

### 2.4. Multi-Agent Successor Representation (SR)

Within the context of SR, given an initial action $a^{\left(i\right)}$ and an initial state $s^{\left(i\right)}$, the expected discounted future state occupancy of state ${s^{\prime }}^{\left(i\right)}$ is estimated based on the current policy $\pi^{\left(i\right)}$ as follows:

(6) $$\begin{array}{c} M_{\pi^{\left(i\right)}}\left(s^{\left(i\right)},{s^{\prime }}^{\left(i\right)},a^{\left(i\right)}\right)=E\left[\sum_{k=0}^{T}\gamma^{k}𝟙\left[s_{k}^{\left(i\right)}={s^{\prime }}^{\left(i\right)}\right]|s_{0}^{\left(i\right)}=s^{\left(i\right)},a_{0}^{\left(i\right)}=a^{\left(i\right)}\right], \end{array}$$

where 𝟙{·}=1 if $s_{k}^{\left(i\right)}={s^{\prime }}^{\left(i\right)}$; otherwise, it is zero. The SR can be represented with a $N_{s^{\left(i\right)}}\times N_{s^{\left(i\right)}}$ matrix when the state-space is discrete. The recursive approach used in Equation (2), can be leveraged to update SR as follows:

(7) $$\begin{array}{cc} M_{\pi^{\left(i\right)}}^{\mathrm{new}} & \left(s_{k}^{\left(i\right)},{s^{\prime }}^{\left(i\right)},a_{k}^{\left(i\right)}\right)=M_{\pi^{\left(i\right)}}^{\mathrm{old}}\left(s_{k}^{\left(i\right)},{s^{\prime }}^{\left(i\right)},a_{k}^{\left(i\right)}\right)+\\ & \alpha \left(𝟙\left[s_{k}^{\left(i\right)}={s^{\prime }}^{\left(i\right)}\right]+\gamma M_{\pi^{\left(i\right)}}\left(s_{k+1}^{\left(i\right)},{s^{\prime }}^{\left(i\right)},a_{k+1}^{\left(i\right)}\right)-M_{\pi^{\left(i\right)}}^{\mathrm{old}}\left(s_{k}^{\left(i\right)},{s^{\prime }}^{\left(i\right)},a_{k}^{\left(i\right)}\right)\right). \end{array}$$

After computation (approximation) of the SR, its inner product with the estimated value of the immediate reward can be used to form the state-action value function based on Equation (1), i.e.,

(8) $$\begin{array}{c} Q_{\pi^{\left(i\right)}}\left(s_{k}^{\left(i\right)},a_{k}^{\left(i\right)}\right)=\sum_{{s^{\prime }}^{\left(i\right)}\in S^{\left(i\right)}}M\left(s_{k}^{\left(i\right)},{s^{\prime }}^{\left(i\right)},a_{k}^{\left(i\right)}\right)R^{\left(i\right)}\left({s^{\prime }}^{\left(i\right)},a_{k}^{\left(i\right)}\right). \end{array}$$

As a final note, it is worth mentioning an important characteristic of the SR-based approach, i.e., the state-action value function can be reconstructed based on the reward function. The developed MARL/MASR formulation presented here is used to develop the proposed MAK-TD/SR frameworks in the following sections.

## 3. The MAK-TD Framework

As stated previously, the MAK-TD framework, is a Kalman-based off-policy learning solution for multi-agent networks. More specifically, by exploiting the TD approach represented in Equation (3), the optimal value function associated with the *i*th agent, for (1≤i≤N), can be approximated from its one-step estimation as follows:

(9) $$\begin{array}{c} Q_{{\pi^{\left(i\right)}}^{*}}\left(s_{k}^{\left(i\right)},a_{k}^{\left(i\right)}\right)\approx r_{k}^{\left(i\right)}+\gamma \max_{a^{\left(i\right)}\in A}Q_{{\pi^{\left(i\right)}}^{*}}\left(s_{k+1}^{\left(i\right)},a^{\left(i\right)}\right). \end{array}$$

By changing the variables’ order, the reward at each time can be represented (modeled) as a noisy observation, i.e.,

(10) $$r_{k}^{\left(i\right)}=Q_{{\pi^{\left(i\right)}}^{*}}\left(s_{k}^{\left(i\right)},a_{k}^{\left(i\right)}\right)-\gamma \max_{a^{\left(i\right)}\in A}Q_{\pi^{*}}\left(s_{k+1}^{\left(i\right)},a^{\left(i\right)}\right)+v_{k}^{\left(i\right)},$$

where $v_{k}$ is modeled as a zero-mean normal distribution with variance of $R^{\left(i\right)}$. By considering the local state-space of each agent, we use localized basis functions to approximate each agent’s value function. Therefore, the following value function can be formed for Agent *i*, for (1≤i≤N),

(11) $$\begin{array}{c} Q_{\pi^{\left(i\right)}}\left(s_{k}^{\left(i\right)},a_{k}^{\left(i\right)}\right)=\phi {\left(s_{k}^{\left(i\right)},a_{k}^{\left(i\right)}\right)}^{T}\theta_{k}^{\left(i\right)}, \end{array}$$

where term $\phi^{\left(i\right)}\left(s^{\left(i\right)},a^{\left(i\right)}\right)$ represents a vector of basis functions, $\pi^{\left(i\right)}$ is the policy associated with Agent *i*, and, finally, $\theta_{k}^{\left(i\right)}$ denotes the vector of the weights. Substituting Equation (11) in Equation (10) results in

(12) $$r_{k}^{\left(i\right)}=\left[\phi {\left(s_{k}^{\left(i\right)},a_{k}^{\left(i\right)}\right)}^{T}-\gamma \max_{a^{\left(i\right)}\in A}\phi {\left(s_{k+1}^{\left(i\right)},a^{\left(i\right)}\right)}^{T}\right]\theta_{k}^{\left(i\right)}+v_{k}^{\left(i\right)},$$

which can be simplified into the following linear observation model:

(13) $$r_{k}^{\left(i\right)}={\left[h_{k}^{\left(i\right)}\right]}^{T}\theta_{k}^{\left(i\right)}+v_{k}^{\left(i\right)},$$

with

(14) $$\begin{array}{c} h_{k}^{\left(i\right)}=\phi \left(s_{k}^{\left(i\right)},a_{k}^{\left(i\right)}\right)-\gamma \max_{a^{\left(i\right)}\in A}\phi \left(s_{k+1}^{\left(i\right)},a^{\left(i\right)}\right). \end{array}$$

In other words, Equation (13) is the localized measurement (reward) of the ith agent, which is a linear model of the weight vector $\theta_{k}^{\left(i\right)}$. For approximating localized weight $\theta_{k}^{\left(i\right)}$, first we leverage the observed reward, which is obtained by transferring from state $s_{k}^{\left(i\right)}$ to $s_{k+1}^{\left(i\right)}$. Second, given that the noise variance of the measurement is not known a priori, we exploit MMAE adaptation by representing it with *M* different values (${R^{j}}^{\left(i\right)}$), for (1≤j≤M). Consequently, a combination of *M* KFs is used to estimate ${\hat{\theta }}_{k}^{\left(i\right)}$ based on each of its candidate values, i.e.,

(15) $$\begin{array}{ccc} {K_{k}^{j}}^{\left(i\right)} & = & P_{\left(\theta ,k|k-1\right)}^{\left(i\right)}h_{k}^{\left(i\right)}{\left(h_{k}^{T^{\left(i\right)}}P_{\left(\theta ,k|k-1\right)}^{\left(i\right)}h_{k}^{\left(i\right)}+{R^{j}}^{\left(i\right)}\right)}^{-1} \end{array}$$

(16) $$\begin{array}{ccc} {\hat{\theta_{k}^{j}}}^{\left(i\right)} & = & {\hat{\theta }}_{\left(k|k-1\right)}^{\left(i\right)}+{K_{k}^{j}}^{\left(i\right)}(r_{k}^{\left(i\right)}-h_{k}^{T^{\left(i\right)}}{\hat{\theta }}_{\left(k|k-1\right)}^{\left(i\right)}) \end{array}$$

(17) $$\begin{array}{ccc} {P_{\theta ,k}^{j}}^{\left(i\right)} & = & (I-{K_{k}^{j}}^{\left(i\right)}h_{k}^{T^{\left(i\right)}}){P_{\left(\theta ,k|k-1\right)}^{T}}^{\left(i\right)}(I-{K_{k}^{j}}^{\left(i\right)}h_{k}^{T^{\left(i\right)}})+{K_{k}^{j}}^{\left(i\right)}{R^{j}}^{\left(i\right)}{{K_{k}^{j}}^{T}}^{\left(i\right)}, \end{array}$$

where superscript *j* is used to refer to the *j*th matched KF, for which a specific value (${R^{j}}^{\left(i\right)}$) is assigned to model covariance of the observation model’s noise process. The posterior distribution associated with each of the *M* matched KFs is calculated based on its likelihood function. All the matched a posteriori distributions are then added together based on their corresponding weights to form the overall posterior distribution given by

(18) $$\begin{array}{c} P^{\left(i\right)}\left(\theta_{k}|Y_{k}\right)=\sum_{j=1}^{M}{\omega^{j}}^{\left(i\right)}P^{\left(i\right)}\left(\theta_{k}^{\left(i\right)}|Y_{k}^{\left(i\right)},{R^{j}}^{\left(i\right)}\right), \end{array}$$

where ${\omega^{j}}^{\left(i\right)}$ is the jth KF’s normalized observation likelihood associated with the ith agent and is given by

(19) $${\omega^{j}}^{\left(i\right)}=P^{\left(i\right)}\left(r_{k}^{\left(i\right)}|\theta_{\left(k|k-1\right)}^{\left(i\right)},R^{j}\right)=c^{\left(i\right)}.e^{[\frac{-1}{2}{\left(r_{k}^{\left(i\right)}-h_{k}^{T^{\left(i\right)}}{\hat{\theta }}_{\left(k|k-1\right)}^{\left(i\right)}\right)}^{T}(h_{k}^{T^{\left(i\right)}}P_{\left(\theta ,k|k-1\right)}^{\left(i\right)}\left(i\right)+{R^{j}}^{\left(i\right)}{)}^{-1}}\left(r_{k}^{\left(i\right)}-{{}^{T}}^{\left(i\right)}{\hat{\theta }}_{\left(k|k-1\right)}^{\left(i\right)})\right],$$

where $c^{\left(i\right)}=1/\left(\sum_{j=1}^{M}{w^{j}}^{\left(i\right)}\right)$. Exploiting Equation (18), the weight and its error covariance are then updated as follows:

(20) $$\begin{array}{ccc} {\hat{\theta }}_{k}^{\left(i\right)} & = & \sum_{j=1}^{M}{\omega^{j}}^{\left(i\right)}\hat{\theta }{{}_{k}^{j}}^{\left(i\right)} \end{array}$$

(21) $$\begin{array}{ccc} P_{\theta ,k}^{\left(i\right)} & = & \sum_{j=1}^{M}{\omega^{j}}^{\left(i\right)}\left({P_{\theta ,k}^{j}}^{\left(i\right)}+\left(\hat{\theta }{{}^{j}}^{\left(i\right)}-{\hat{\theta }}^{\left(i\right)}\right){\left(\hat{\theta }{{}^{j}}^{\left(i\right)}-{\hat{\theta }}^{\left(i\right)}\right)}^{T}\right). \end{array}$$

To finalize computation of ${\hat{\theta }}_{k}^{\left(i\right)}$ based on Equations (13)–(20), localized measurement mapping function $h_{k}^{\left(i\right)}$ is required. As $h_{k}^{\left(i\right)}$ is formed by the basis functions, its adaptation necessitates the adaptation of the basis functions. The vector of basis functions shown in Equation (11) is formed as follows:

(22) $$\phi \left(s_{k}^{\left(i\right)}\right)={\left[\phi_{1}\left(s_{k}^{\left(i\right)}\right),\phi_{2}\left(s_{k}^{\left(i\right)}\right),\ldots ,\phi_{N_{b}-1}\left(s_{k}^{\left(i\right)}\right),\phi_{N_{b}}\left(s_{k}^{\left(i\right)}\right)\right]}^{T},$$

where $N_{b}$ is the number of basis functions. Each basis function is represented by a RBF, which is defined by its mean and covariance parameters as follows:

(23) $$\phi_{n}\left(s_{k}^{\left(i\right)}\right)=\exp\left\{\frac{-1}{2}{\left(s_{k}^{\left(i\right)}-\mu_{n}^{\left(i\right)}\right)}^{T}{\Sigma_{n}^{\left(i\right)}}^{-1}\left(s_{k}^{\left(i\right)}-\mu_{n}^{\left(i\right)}\right)\right\},$$

where $\mu_{n}^{\left(i\right)}$ and $\Sigma_{n}^{\left(i\right)}$ are the mean and covariance of $\phi_{n}\left(s_{k}^{\left(i\right)}\right)$, for ($1\leq n\leq N_{b}$). Generally speaking, the state-action feature vector can be represented as follows:

(24) $$\begin{array}{c} \phi \left(s_{k}^{\left(i\right)},a_{k}^{\left(i\right)}\right)={\left[\phi_{1,a_{1}}\left(s_{k}^{\left(i\right)}\right),\ldots \phi_{N_{b},a_{1}}\left(s_{k}^{\left(i\right)}\right),\phi_{1,a_{2}}\left(s_{k}^{\left(i\right)}\right),\ldots \phi_{N_{b},a_{D^{\left(i\right)}}}\left(s_{k}^{\left(i\right)}\right)\right]}^{T}, \end{array}$$

where $\phi \left(\cdot \right):A^{\left(i\right)}\times S\to R^{N_{b}\times D^{\left(i\right)}}$, and $D^{\left(i\right)}$ denotes the number of actions associated with the ith agent. The state-action feature vector $\phi (s_{k}^{\left(i\right)},a_{k}^{\left(i\right)}=a_{d}^{\left(i\right)})$, for ($1\leq d\leq D^{\left(i\right)}$) in Equation (24) is considered to be generated from $\phi (s_{k}^{\left(i\right)})$ by placing this state feature vector in the corresponding spot for action $a_{k}^{\left(i\right)}$ while the feature values for the rest of the actions are set to zero, i.e.,

(25) $$\phi \left(s_{k}^{\left(i\right)},a_{k}^{\left(i\right)}\right)={\left[0,\ldots 0,\phi_{1}\left(s_{k}^{\left(i\right)}\right),\ldots ,\phi_{N}\left(s_{k}^{\left(i\right)}\right),0,\ldots 0,\right]}^{T}.$$

Due to the large number of parameters associated with the measurement mapping function, the multiple model approach seems to be inapplicable. Alternatively, Restricted Gradient Descent (RGD) [32] is employed, where the goal is to minimize the following loss function:

(26) $$L_{k}^{\left(i\right)}={\left(\phi^{T}\left(s_{k}^{\left(i\right)},a_{k}\right)\;\theta_{k}^{\left(i\right)}-r_{k}^{\left(i\right)}\right)}^{2}.$$

The gradient of the objective function with respect to the parameters of each basis function is then calculated using the chain rule as follows:

(27) $$\begin{array}{ccc} \Delta \mu^{\left(i\right)} & = & -\frac{\partial L_{k}^{\left(i\right)}}{\partial \mu^{\left(i\right)}}=-\frac{\partial L_{k}^{\left(i\right)}}{\partial Q_{{\pi^{*}}^{\left(i\right)}}}\frac{\partial Q_{{\pi^{*}}^{\left(i\right)}}}{\partial \phi^{\left(I\right)}}\frac{\partial \phi^{\left(I\right)}}{\partial \mu^{\left(i\right)}} \end{array}$$

(28) $$\begin{array}{ccc} \mathrm{and}\;\Delta \Sigma^{\left(i\right)} & = & -\frac{\partial \Sigma_{k}^{\left(i\right)}}{\partial \mu^{\left(i\right)}}=-\frac{\partial L_{k}^{\left(i\right)}}{\partial Q_{{\pi^{*}}^{\left(i\right)}}}\frac{\partial Q_{{\pi^{*}}^{\left(i\right)}}}{\partial \phi^{\left(I\right)}}\frac{\partial \phi^{\left(I\right)}}{\partial \Sigma^{\left(i\right)}}, \end{array}$$

where calculation of the partial derivations is done leveraging Equations (11), (23) and (26). Therefore, the mean and covariance of the RBFs can be adapted using the calculated partial derivative as follows:

(29) $$\begin{array}{ccc} \mu_{n}^{\left(i\right)} & = & \mu_{n}^{\left(i\right)}-2\lambda_{\mu^{\left(i\right)}}{\left(L_{k}^{\left(i\right)}\right)}^{\frac{1}{2}}{\theta_{k}^{\left(i\right)}}^{T}{\left(\Sigma_{n}^{\left(i\right)}\right)}^{-1}\left(s_{k}^{\left(i\right)}-\mu_{n}^{\left(i\right)}\right) \end{array}$$

(30) $$\begin{array}{ccc} \Sigma_{n}^{\left(i\right)} & = & \Sigma_{n}^{\left(i\right)}-2\lambda_{\Sigma^{\left(i\right)}}{\left(L_{k}^{\left(i\right)}\right)}^{\frac{1}{2}}{{\theta^{\left(i\right)}}_{k}}^{T}{\left(\Sigma_{n}^{\left(i\right)}\right)}^{-1}\times \left(s_{k}^{\left(i\right)}-\mu_{n}^{\left(i\right)}\right){\left(s_{k}^{\left(i\right)}-\mu_{n}^{\left(i\right)}\right)}^{T}{\Sigma_{n}^{\left(i\right)}}^{-1}, \end{array}$$

where both $\lambda_{\mu^{\left(i\right)}}$ and $\lambda_{\Sigma^{\left(i\right)}}$ denote the adaptation rates. Based on [32], for the sake of stability, only one of the updates shown in Equations (29) and (30), will be applied. To be more precise, when the size of the covariance is decreasing (i.e., ${L_{k}^{\left(i\right)}}^{\frac{1}{2}}\left({\theta_{k}^{\left(i\right)}}^{T}\phi \left(\cdot \right)\right)>0$), the covariances of the RBFs are updated using Equation (30); otherwise, their means are updated using Equation (29). Using this approach, unlimited expansion of the RBF covariances is avoided.

One superiority that the proposed learning framework shows over other optimization-based techniques (e.g., gradient descent-based methods) is the calculation of the uncertainty for the weights $P_{\theta ,k}^{\left(i\right)}$, which is directly related to the uncertainty of the value function. This information can then be used at each step to select the actions, leading to the most reduction in the weights’ uncertainty. Using the information form of the KF (information filter [59]), the information of the weights denoted by $P_{\theta ,k}^{\left(i\right)}$ is updated as follows:

(31) $$\begin{array}{c} {P_{\theta ,k}^{-1}}^{\left(i\right)}={P_{\left(\theta {,}_{k|k-1}\right)}^{-1}}^{\left(i\right)}{+}^{\left(i\right)}{R^{-1}}^{\left(i\right)}{{}^{T}}^{\left(i\right)}. \end{array}$$

In Equation (31), the second element, i.e., ${}^{\left(i\right)}{R^{-1}}^{\left(i\right)}{{}^{T}}^{\left(i\right)}$, represents the information received from the measurement. The action is obtained by maximizing the information of the weights, i.e.,

(32) $$\begin{array}{ccc} a_{k}^{\left(i\right)} & = & \arg\max_{a}\left(h_{k}^{\left(i\right)}\left(s_{k}^{\left(i\right)},a^{\left(i\right)}\right){R^{-1}}^{\left(i\right)}h_{k}^{T^{\left(i\right)}}\left(s_{k}^{\left(i\right)},a^{\left(i\right)}\right)\right)\\ & = & \arg\max_{a}\left(h_{k}^{\left(i\right)}\left(s_{k}^{\left(i\right)},a^{\left(i\right)}\right)h_{k}^{T^{\left(i\right)}}\left(s_{k}^{\left(i\right)},a^{\left(i\right)}\right)\right). \end{array}$$

The second equality in Equation (32) is constructed as $R^{\left(i\right)}$ is a scalar. The projected behavior policy in Equation (32) is different from that in [37], where a random policy was proposed, which favored actions with less certainty of the value function. Although reducing the value function’s uncertainty through action selection is an intelligent approach, it is less efficient in sample selection due to the random nature of such policies. Algorithm 1 briefly represents the MAK-TD framework proposed in this work.
**Algorithm 1**The Proposed MAK-TD Framework1:**Learning Phase:**2:Set $\theta_{0},P_{\theta ,0},F,\mu_{n,i_{d}},\Sigma_{n,i_{d}}$ for n=1,2,⋯,N and $i_{d}=1,2,\cdots ,D$3:**Repeat** (for each episode):4:      Initialize $s_{k}$5:      **Repeat** (for each agent *i*):6:         **While** $s_{k}^{\left(i\right)}\neq s_{T}$ **do**:7:            $a_{k}^{\left(i\right)}=\arg\max_{a}\left(h_{k}^{\left(i\right)}\left(s_{k}^{\left(i\right)},a^{\left(i\right)}\right)h_{k}^{T^{\left(i\right)}}\left(s_{k}^{\left(i\right)},a^{\left(i\right)}\right)\right)$8:            Take action $a_{k}^{\left(i\right)}$, observe $s_{k+1}^{\left(i\right)},r_{k}^{\left(i\right)}$9:            Calculate $\phi^{\left(I\right)}\left(s^{\left(i\right)},a^{\left(i\right)}\right)$ via Equations (22) and (23)10:            $h_{k}^{\left(i\right)}\left(s_{k}^{\left(i\right)},a_{k}^{\left(i\right)}\right)=\phi^{\left(I\right)}\left(s_{k}^{\left(i\right)},a_{k}^{\left(i\right)}\right)-\gamma \arg\max_{a}\phi^{\left(I\right)}\left(s_{k+1}^{\left(i\right)},a^{\left(i\right)}\right)$11:            ${\hat{\theta }}_{\left(k|k-1\right)}^{\left(i\right)}=F^{\left(i\right)}{{\hat{\theta }}_{k}}^{\left(i\right)}$12:            $P_{\left(\theta ,k|k-1\right)}^{\left(i\right)}=F^{\left(i\right)}P_{\theta ,k-1}^{\left(i\right)}{F^{T}}^{\left(i\right)}+Q^{\left(i\right)}$13:            **for** j=1:M **do**:14:                  ${K_{k}^{j}}^{\left(i\right)}=P_{\left(\theta ,k|k-1\right)}^{\left(i\right)}h_{k}^{\left(i\right)}{\left(h_{k}^{T^{\left(i\right)}}P_{\left(\theta ,k|k-1\right)}^{\left(i\right)}h_{k}^{\left(i\right)}+{R^{j}}^{\left(i\right)}\right)}^{-1}$15:                  $\hat{\theta }{{}_{k}^{j}}^{\left(i\right)}={\hat{\theta }}_{\left(\theta ,k|k-1\right)}^{\left(i\right)}+{K_{k}^{j}}^{\left(i\right)}\left(r_{k}^{j}{-}^{\left(i\right)}{\hat{\theta }}_{\left(k|k-1\right)}^{\left(i\right)}\right)$16:                   $P_{\theta ,k}^{\left(i\right)}=\left(I-{K_{k}^{j}}^{\left(i\right)}h_{k}^{T^{\left(i\right)}}\right)P_{\left(\theta ,k|k-1\right)}^{\left(i\right)}{\left(I-{K_{k}^{j}}^{\left(i\right)}h_{k}^{T^{\left(i\right)}}\right)}^{T}+{K_{k}^{j}}^{\left(i\right)}R^{j}{{K_{k}^{j}}^{T}}^{\left(i\right)}$17:            **end for**18:             Compute the value of *c* and ${w^{j}}^{\left(i\right)}$ by using $\sum_{j=1}^{M}{w^{j}}^{\left(i\right)}=1$ and Equation (19)19:            ${\hat{\theta }}_{k}^{\left(i\right)}=\sum_{j=1}^{M}{w^{j}}^{\left(i\right)}\hat{\theta }{{}_{k}^{j}}^{\left(i\right)}$20:            $P_{\theta_{k}}^{\left(i\right)}=\sum_{j=1}^{M}{\omega^{j}}^{\left(i\right)}\left({P_{\theta {,}_{k}}^{j}}^{\left(i\right)}+\left(\hat{\theta }{{}^{j}}^{\left(i\right)}-{\hat{\theta }}^{\left(i\right)}\right){\left(\hat{\theta }{{}^{j}}^{\left(i\right)}-{\hat{\theta }}^{\left(i\right)}\right)}^{T}\right)$21:            **RBFs Parameters Update:**22:            $L_{k}^{\left(i\right)}={\left(\phi^{T}\left(s_{k}^{\left(i\right)},a_{k}\right)\;\theta_{k}^{\left(i\right)}-r_{k}^{\left(i\right)}\right)}^{2}$23:            **if** ${L_{k}^{\left(i\right)}}^{\frac{1}{2}}\left({\theta_{k}^{\left(i\right)}}^{T}\phi \left(\cdot \right)\right)>0$ **then**:24:               Update $\Sigma_{n,a_{d}}$ via Equation (29)25:            **else:**26:               Update $\mu_{n,a_{d}}$ via Equation ()27:            **end if**28:         **end while**29:**Testing Phase:**30:**Repeat** (for each trial episode):31:      **While** $s_{k}\neq s_{T}$ **do**:32:         **Repeat** (for each agent):33:            $a_{k}=\arg\max_{a}\phi {\left(s_{k},a\right)}^{T}\theta_{k}$34:            Take action $a_{k}$, and observe $s_{k+1},r_{k}$35:            Calculate Loss $s_{k}$ for all agents36:      **End While**

## 4. The MAK-SR Framework

In the previous section, the MAK-TD framework is proposed, which is a MM Kalman-based off-policy learning solution for multi-agent networks. To learn the value function, a fixed model for the reward function is considered, which could restrict its application to more complex MARL problems. SR-based algorithms are appealing solutions to tackle this issue where the focus is instead on learning the immediate reward and the SR, which is the expected discounted future state occupancy. In the existing SR-based approaches that use standard temporal difference methods, the uncertainty about the approximated SR is not captured. In order to address this issue, we extend the MAK-TD framework and design its SR-based variant in this section. In other words, MAK-TD is extended to MAK-SR by incorporation of the SR learning procedure into the filtering problem using KTD formulation to estimate uncertainty of the learned SR. Moreover, by applying KTD, we benefit from the decrease in memory and time spent for the SR learning and also sensitivity of the framework’s performance to its parameters (i.e., more reliable) when compared to DNN-based algorithms.

Exact computation of the SR and the reward function is, typically, not possible within the multi-agent settings as we are dealing with a large number of continuous states. Therefore, we follow the approach developed in Section 3 and approximate the SR and the reward function via basis functions. For the state-action feature vector $\phi (s^{\left(i\right)},a^{\left(i\right)})$, a feature-based SR, which encodes the expected occupancy of the features, is defined as follows:

(33) $$M_{\pi^{\left(i\right)}}\left(s^{\left(i\right)},:,a^{\left(i\right)}\right)=E\left[\sum_{k=0}^{T}\gamma^{k}\phi \left(s_{k}^{\left(i\right)},a_{k}^{\left(i\right)}\right)|s_{0}^{\left(i\right)}=s^{\left(i\right)},a_{0}^{\left(i\right)}=a^{\left(i\right)}\right].$$

We consider that the immediate reward function for pair $\left(s^{\left(i\right)},a^{\left(i\right)}\right)$ can be linearly factorized as

(34) $$\begin{array}{c} r^{\left(i\right)}\left(s_{k}^{\left(i\right)},a_{k}^{\left(i\right)}\right)\approx \phi {\left(s_{k}^{\left(i\right)},a_{k}^{\left(i\right)}\right)}^{T}\theta_{k}^{\left(i\right)}, \end{array}$$

where $\theta_{k}^{\left(i\right)}$ is the reward weight vector. The state-action value function (Equation (8)), therefore, can be computed as follows:

(35) $$\begin{array}{c} Q\left(s_{k}^{\left(i\right)},a_{k}^{\left(i\right)}\right)={\theta_{k}^{\left(i\right)}}^{T}M\left(s_{k}^{\left(i\right)},:,a_{k}^{\left(i\right)}\right). \end{array}$$

The SR matrix $M(s_{k}^{\left(i\right)},:,a_{k}^{\left(i\right)})$ can be approximated as a linear function of the same feature vector as follows:

(36) $$\begin{array}{c} M_{\pi^{\left(i\right)}}\left(s_{k}^{\left(i\right)},:,a_{k}^{\left(i\right)}\right)\approx M_{k}\;\phi \left(s_{k}^{\left(i\right)},a_{k}^{\left(i\right)}\right). \end{array}$$

The TD learning of the SR then can be performed as follows:

(37) $$M_{\pi^{\left(i\right)}}^{\mathrm{new}}\left(s_{k}^{\left(i\right)},:,a_{k}^{\left(i\right)}\right)=M_{\pi^{\left(i\right)}}^{\mathrm{old}}\left(s_{k}^{\left(i\right)},:,a_{k}^{\left(i\right)}\right)+\alpha \left(\phi^{\left(i\right)}\left(s_{k}^{\left(i\right)},a_{k}^{\left(i\right)}\right)+\gamma M_{\pi^{\left(i\right)}}\left(s_{k+1}^{\left(i\right)},:,a_{k+1}^{\left(i\right)}\right)-M_{\pi^{\left(i\right)}}^{\mathrm{old}}\left(s_{k}^{\left(i\right)},:,a_{k}^{\left(i\right)}\right)\right).$$

By defining the estimation structure of the SR and reward function, a suitable method must be selected to learn (approximate) the weight vector of the reward $\theta^{\left(i\right)}$ and the weight matrix of the SR M for Agent *i*. The proposed multi-agent MAK-SR algorithm contains two main components: KTD-based weight SR learning and radial basis function update. For the latter, we apply the method developed in Section 3 to approximate the vector of basis functions via representing each of them as a RBF. The gradient of the loss function (26), with respect to the parameters of the RBFs, is calculated using the chain rule for the mean and covariance of RBFs using (29) and (30).

For KTD-based weight SR learning, the SR can be obtained from its one-step approximation using the TD method of Equation (37). In this regard, the state-action feature vector at time step *k* can be considered as a noisy measurement from the system as follows:

(38) $$\hat{\phi }\left(s_{k}^{\left(i\right)},a_{k}^{\left(i\right)}\right)=M^{\mathrm{new}}\left(s_{k}^{\left(i\right)},:,a_{k}^{\left(i\right)}\right)-\gamma M\left(s_{k+1}^{\left(i\right)},:,a_{k+1}^{\left(i\right)}\right)+n_{k}^{\left(i\right)},$$

where $n_{k}^{\left(i\right)}$ follows a zero-mean normal distribution with covariance of $R_{M}^{\left(i\right)}$. Considering Equations (36) and (38) together, the feature vector $\phi (s_{k}^{\left(i\right)},a_{k}^{\left(i\right)})$ can be approximated as

(39) $$\hat{\phi }\left(s_{k}^{\left(i\right)},a_{k}^{\left(i\right)}\right)=M_{k}\underset{g_{k}^{\left(i\right)}}{\underset{︸}{\left[\phi \left(s_{k}^{\left(i\right)},a_{k}^{\left(i\right)}\right)-\gamma \phi \left(s_{k+1}^{\left(i\right)},a_{k+1}^{\left(i\right)}\right)\right]}}+n_{k}^{\left(i\right)}.$$

s Matrix $M_{k}$ is then mapped to a column vector $M_{k}^{\left(i\right)}$ by concatenating its columns. Using the vec-trick characteristic of Kronecker product denoted by ⊗, then we can rewrite Equation (39) as follows:

(40) $$\begin{array}{c} \hat{\phi }\left(s_{k}^{\left(i\right)},a_{k}^{\left(i\right)}\right)=\left({g^{\left(i\right)}}_{k}^{T}⊗I\right)M_{k}^{\left(i\right)}+n_{k}^{\left(i\right)}, \end{array}$$

where I represents an identity matrix of appropriate dimension. More specifically, Equation (40) is used to represent the localized measurements ($\phi (s_{k}^{\left(i\right)},a_{k}^{\left(i\right)})$) linearly based on vector $M_{k}^{\left(i\right)}$, which requires estimation. Therefore, we use the following linear state model:

(41) $$\begin{array}{c} M_{k+1}^{\left(i\right)}=M_{k}^{\left(i\right)}+\mu_{k}^{\left(i\right)}, \end{array}$$

to complete the required state-space representation for KF-based implementation. The noise associated with the state model (Equation (41)), i.e., $\mu_{k}^{\left(i\right)}$, follows a zero-mean normal distribution with covariance of $Q_{M}$. Via implementing the KF’s recursive equations, we use the new localized observations to estimate $M_{k}^{\left(i\right)}$ and its corresponding covariance matrix $P_{M^{\left(i\right)},k}^{\left(i\right)}$. After this step, vector $M_{k}^{\left(i\right)}$ is reshaped to form a (L×L) matrix in order to reconstruct Matrix $M_{k}$. Equation (35) is finally used to form the state-action value function for associated with ($s_{k}^{\left(i\right)},a_{k}^{\left(i\right)}$). Algorithm 2 summarizes the proposed MAK-SR framework.
**Algorithm 2**The Proposed MAK-SR Framework1:**Learning Phase:**2:**Initialize:**$\theta_{0},P_{\theta ,0},M_{0},P_{M,0},\mu_{n},\mathrm{and}\;\Sigma_{n}$ for n=1,2,⋯,N3:**Parameters:**$Q_{\theta },Q_{M},\lambda_{\mu },\lambda_{\Sigma },\mathrm{and}\;\left\{R_{\theta }^{j},R_{M}^{j}\right\}$ for j=1,2,⋯,M4:**Repeat** (for each episode):5:   Initialize $s_{k}$6:   **Repeat** (for each agent *i*):7:      **While** $s_{k}^{\left(i\right)}\neq s_{T}$ **do**:8:         Reshape $M_{k}$ into L×L to construct 2-D matrix $M_{k}$.9:         $a_{k}^{\left(i\right)}=\arg\max_{a}\left(g_{k}^{\left(i\right)}\left(s_{k}^{\left(i\right)},a\right){g_{k}^{\left(i\right)}}^{T}\left(s_{k}^{\left(i\right)},a^{\left(i\right)}\right)\right)$10:         Take action $a_{k}^{\left(i\right)}$, observe $s_{k+1}^{\left(i\right)}$ and $r_{k}^{\left(i\right)}$.11:         Calculate $\phi (s_{k}^{\left(i\right)},a_{k}^{\left(i\right)})$ via Equations (23) and (25).12:         **Update reward weights vector:** Perform MMAE to update $\theta_{k}^{\left(i\right)}$.13:         **Update SR weights vector:** Perform KF on Equations (40) and (41) to update $M_{k}^{\left(i\right)}$.14:         **Update RBFs parameters:** Perform RGD on the loss function $L_{k}$ to update $\Sigma_{n}$ and $\mu_{n}$.15:      **end while**

It is worth mentioning that, unlike the DNN-based networks for multi-agent scenarios, the proposed multiple-model frameworks require far less memory due to their sequential data processing nature. In other words, storing the whole episodes’ information for all the agents is not needed as the last measured data (assuming one-step Markov decision process) can be leveraged given the sequential nature of the incorporated filters. Finally, note that the proposed MAK-SR and MAK-TD frameworks are designed for systems with a finite number of actions. One direction for future research is to consider extending the proposed MAK-SR framework to applications where the to action-space is infinite-dimensional. This might occur in continuous control problems [54,60] where number of possible actions at each state is infinite.

## 5. Experimental Results

**Figure 1:**
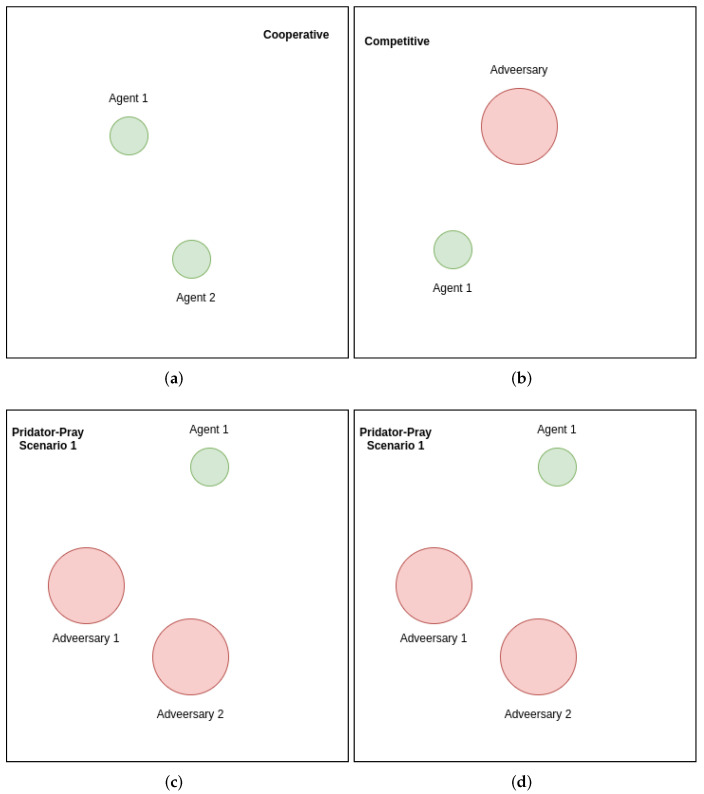
Different multi-agent scenarios implemented within the OpenAI gym. (**a**) Cooperation Scenario (**b**) Competition Scenario (**c**) Predator-Prey 2v1, and (**d**) Predator-Prey 1v2.

The performances of the proposed MAK-SR and MAK-TD frameworks are evaluated in this section, where a multi-agent extension of the OpenAI gym benchmark is utilized. Figure 1 illustrates snapshots of the environment utilized for evaluation of the proposed approaches. More specifically, a two-dimensional world is implemented to simulate competitive, cooperative, and/or mix interaction scenarios [50]. The utilized benchmark is currently one of the most standard environments to test different multi-agent algorithms, where time, discrete action space, and continuous observations are the basics of the environment. Such a multi-agent environment is a natural curriculum in that the environment difficulty is determined based on the skills of the agents cooperating or competing. The environment does not have a stable equilibrium, therefore, allowing the participating agents to become smarter irrespective of their intelligence level. In each step, the implemented environment provides observations and rewards once the agents performed their actions. The proposed platforms are implemented on a computer with a 3.79 GHz AMD Ryzen 9, 12-core processor. The frameworks are evaluated via several experiments, which are implemented through the OpenAI Gym multi-agent RL benchmarks. The parameters related to the proposed MAK-SR and MAK-TD are set randomly. In the designed deep models, the learning rate is set as 0.001, and the models are trained with the mini-batches of size 128 using Adam Optimizer. MADDPG and DDPG are based on the Actor-Critic approach. DQN and DDPG receive an observation as input consisting of the current state, next state, gained reward, and the action taken by the agents at each step in the environment. For MADDPG, based on the received state data (current and next state) and the actions taken by all the agents, the future return is approximated considering all the agent’s policies.

In what follows, we discuss different multi-agent environments exploited in this work as well as the experimental assumptions considered during testing of the proposed methods. Finally, the results of the experiments will be represented and explained.

### 5.1. Environments

In the represented multi-agent environments, we do not impose any assumption or requirement on having identical observations or action spaces for the agents. Furthermore, agents are not restricted to follow the same policy π while playing the game. In the environments, a different number of agents and possible landmarks can be placed to establish different interactions such as cooperative, competitive, or mixed strategies. The strategy in each environment is to keep the agents in the game as long as possible. Each test can be fully cooperative when agents communicate to maximize a shared return, or can be fully competitive when the agents compete to achieve different goals. The mixed scenario for the predator–prey environments (a variant of the classical predator–prey) is defined in a way that a group of slower agents must cooperate against another group of faster agents to maximize their returned reward. Each agent takes a step by choosing one of five available actions, i.e., no movement, left, right, up, and down, transiting to a new state, and receiving a reward from the environment. Moreover, each agent will receive a list of observations in each state, which contains the agent’s position and velocity, relative positions of landmarks (if available), and its relative position to other agents in the environment. That is how an agent knows the position and general status of the agents (friends and adversaries), enabling the decision-making process of that agent. As shown in Figure 1, each environment has its own margins. An agent that leaves the area will be punished by −50 points, the game will be reset, and a random configuration will be initiated to start the next state, which begins immediately. The red agents play the predator role and receive +100 points intercepting (hunting) a prey (small green agents). The green agents that are faster than red agents (predators) will receive −100 points by each interception with the red ones. As their job is to follow the prey, the predators will be punished proportionally to their distance to the prey (green agents). In contrast, the opposite will happen to the green agents as they keep the maximum distance from the predators. The proposed MAK-TD/SR frameworks are evaluated against DQN [26], DDPG [27], and MADDPG [57]. We evaluate the algorithms in terms of loss, returned discounted reward, and the number of collisions between agents.

### 5.2. Experimental Assumptions

In the proposed frameworks, we exploit related RBFs based on the different agents’ sizes of observations and a bias parameter. The size of the observation vector at each local agent (localized observation vector), which represents the number of global and local measurements available locally, varies across different scenarios based on the type and the number of agents present/active in the environment. Irrespective of size of the localized observation vectors, the size of the localized feature vectors, which represents the available five actions, is considered to be 50. Mean and covariance of the RBFs are initialized randomly for all the agents in all the environments. For example, consider a Predator–Prey scenario with 2 preys optimizing their actions against one predator. In this toy-example (discussed for clarification purposes), considering 9 RBFs together with localized observation vectors of size 12 for the predator and 10 for the preys, the mean vector associated with the predator and the preys are of dimensions 9×12 and 9×10, respectively. Consequently, for this Predator–Prey scenario, μ, which is initialized randomly contains three agents with random values with the mean size ((9,12),(9,10),(9,10)) and the covariance, $\Sigma =(I_{12},I_{10},I_{10})$ where $I_{12}$ and $I_{10}$ are the identity matrices of size (12×12) and (10×10), respectively. Based on Equation (25), the vector of basis function is represented as follows:

(42) $$\begin{array}{ccc} \phi (s_{k},a_{k}=-2) & = & {\left[0,\ldots ,0,0,\ldots ,0,1,\phi_{1,a_{d}},\ldots \phi_{9,a_{d}},0,\ldots ,0,0,\ldots ,0\right]}^{T}, \end{array}$$

(43) $$\begin{array}{ccc} \phi (s_{k},a_{k}=-1) & = & {\left[0,\ldots ,0,0,\ldots ,0,0,\ldots ,0,1,\phi_{1,a_{d}},\ldots \phi_{9,a_{d}},0,\ldots ,0\right]}^{T}, \end{array}$$

(44) $$\begin{array}{ccc} \phi (s_{k},a_{k}=0) & = & {\left[0,\ldots ,0,0,\ldots ,0,0,\ldots ,0,0,\ldots ,0,1,\phi_{1,a_{d}},\ldots \phi_{9,a_{d}}\right]}^{T}, \end{array}$$

(45) $$\begin{array}{ccc} \phi (s_{k},a_{k}=+1) & = & {\left[0,\ldots ,0,1,\phi_{1,a_{d}},\ldots ,\phi_{9,a_{d}},0,\ldots 0,0,\ldots ,0,0,\ldots ,0\right]}^{T} \end{array}$$

(46) $$\begin{array}{ccc} \mathrm{and}\;\phi (s_{k},a_{k}=+2) & = & {\left[1,\phi_{1,a_{d}},\ldots ,\phi_{9,a_{d}},0,\ldots ,0,0,\ldots ,0,0,\ldots 0,0,\ldots 0\right]}^{T}, \end{array}$$

where $\phi_{l,a_{d}}$ is calculated based on Equation (24) for (l∈{1,2,⋯,9}., γ, In all the scenarios, the time step chosen to be 10 milliseconds and the discount factor is 0.95. The transition matrix is initiated to $F=I_{50}$, and for the process noise covariance, a small value of $Q_{k}=10^{-7}I_{50}$ is considered. The covariance matrix associated with the noise of the measurement model is selected from the following set:

(47) $$\begin{array}{c} R^{\left(i\right)}\in \left\{0.01,0.1,0.5,1,5,10,50,100\right\}. \end{array}$$

For initializing the weights, we sample from a zero mean Gaussian initialization distribution $N(\theta_{0},P_{\theta ,0})$, where $\theta_{0}=0_{50}$ and $P_{\theta ,0}=10I_{50}$. By considering the aforementioned initial parameters, each experiment is initiated randomly and consists of 1000 learning episodes together with 1000 test episodes. Given small number of available learning episodes, the proposed MAK-TD/SR frameworks outperformed their counterparts across different metrics including sample efficiency, cumulative reward, cumulative steps, and speed of the value function convergence.

### 5.3. Results

**Figure 2:**
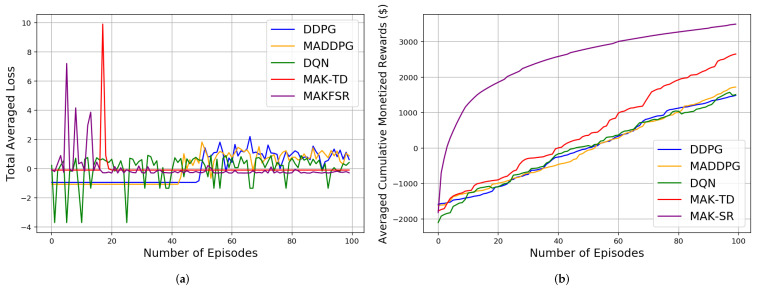
The Predator–Prey environment: (**a**) Loss. (**b**) Received rewards.

**Figure 3:**
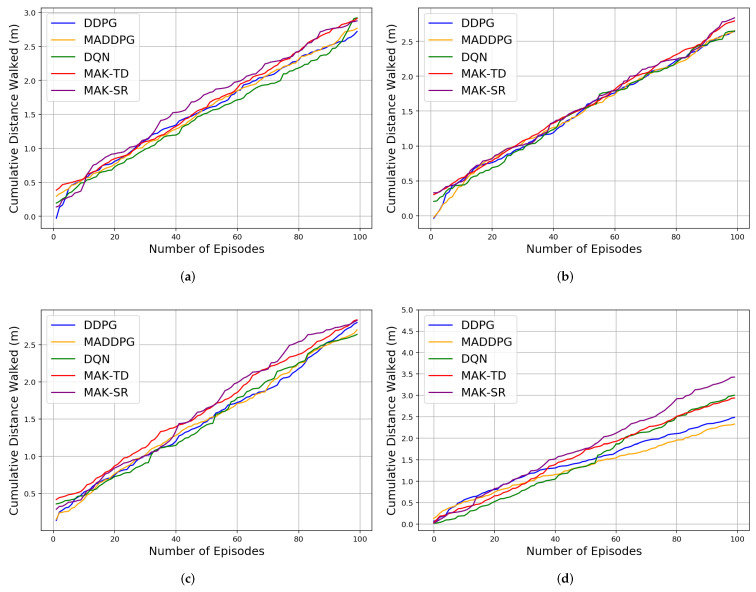
Cumulative distance walked by the agents in four different environments based on the five implemented algorithms (**a**) Cooperation. (**b**) Competition. (**c**) Predator–Prey 2v1. (**d**) Predator–Prey 1v2.

**Figure 4:**
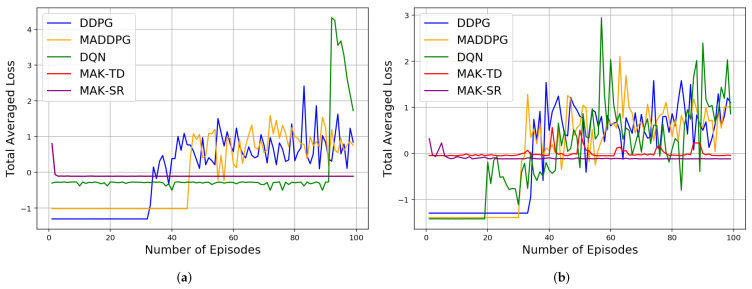

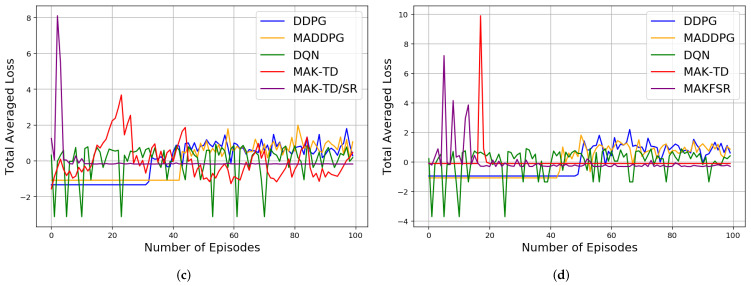
Four different normalized loss functions results for all the agents in the for the four algorithms in four different environments: (**a**) Cooperation. (**b**) Competition. (**c**) Predator–Prey 2v1. (**d**) Predator–Prey 1v2.

Initially, the agents are trained over different number of episodes, after which 10 iteration each of 1000 episodes is implemented for testing to compute different results evaluating performance and efficiency of the proposed MAK-TD/SR frameworks. First, to evaluate stability of the incorporated RBFs, a Monte Carlo (MC) study is conducted where 10 RBFs are used across all the environments. The results are averaged over multiple realizations leveraging MC sampling as shown in Table 1, Table 2 and Table 3. Figure 2b shows the rewards gained by all the agents in a Predator–Prey environment. It is worth mentioning that the average number of the steps taken by all the agents in the defined environments is also represented in Table 3, showing MAK-SR remarkable results in contrast with the other algorithms. Results related to cumulative distance walked by the agents (computed by multiplying the number of the steps by 0.74 m for each step) are also shown in Figure 3 for different environments admitting superiority of the MAK-SR framework in contrast with other solutions. The loss function associated with each of the five implemented methods is shown in Figure 4.

## 6. Discussion

The results shown in Section 5 illustrate the inherent stability of the utilized RBFs and the proposed MAK-TD and MAK-SR frameworks. Capitalizing on the results of Table 1, Table 2 and Table 3, the MAK-SR can be considered as the most sample-efficient approach. It is worth noting that although MAK-SR outperforms the MAK-TD approach, we included both, as the learned representation is not transferable between optimal policies in the SR learning. For such scenarios, MAK-TD is an alternative solution providing, more or less, similar performance to that of the MAK-SR. To be more precise, when solving a previously unseen MDP, a learned SR representation can only be used for initialization. In other words, the agents still have to adjust the SR representation to the policy, which is only optimal within the existing MDP. This limitation urges us to represent the MAK-TD as another trusted solution.

As it can be seen from Table 1, the average loss associated with the proposed MAK-SR is better than that of the MAK-TD. Both frameworks, however, outperform their counterparts, which can be attributed to their improved sample selection efficiency. This excellence can also be seen for the Predator–Prey 1v2 environment in Figure 2a. The calculated losses mostly have small values after the beginning of the experiments, indicating stability of the implemented frameworks. As can be seen, other approaches cannot provide that level of performance that is achieved by MAK-SR and MAK-TD with such low number of training episodes in this experiment. The other three DNN-based approaches can reach such an efficiency with a much greater amount of experience (more than 10,000 experiments) and use much more memory space to save the batches of the information.

**Figure 5:**
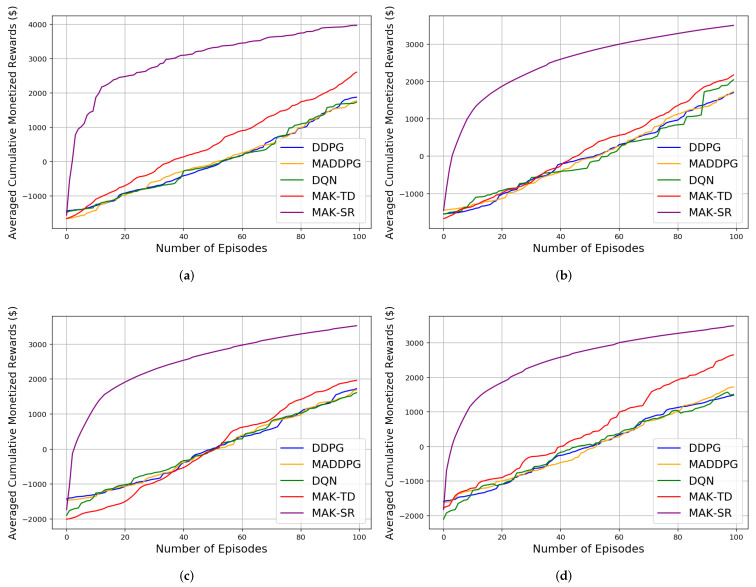
Four different reward functions results for all the agents for the five algorithms in four different environments: (**a**) Cooperation. (**b**) Competition. (**c**) Predator–Prey 2v1. (**d**) Predator–Prey 1v2.

As can be seen in Table 2 and Figure 2b, the rewards gained in the MAK-SR are also better than those of the MAK-TD and are much higher than the other approaches. This can be considered exceptional considering the limited utilized experience. For all other environments, this better performance in the gained reward can be seen in Figure 5 where four different reward functions for five discussed algorithms in four experiment environments are shown. As expected, the performance of each model improves over time as being trained through different training episodes. The proposed MAK-SR and MAK-TD provide exceptional performances given the small number of training episodes utilized in these experiments. MADDPG, DDPG, and DQN, however, fail to achieve the same performance level.

**Figure 6:**
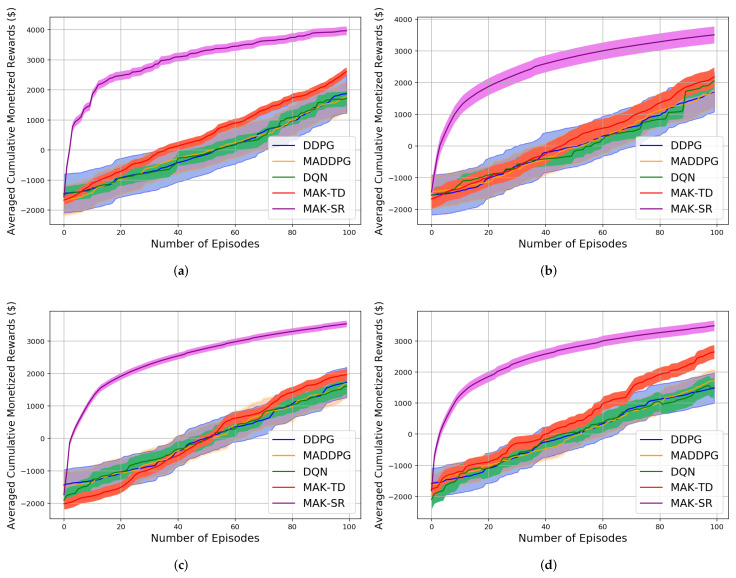
The mean (solid lines) and standard deviation (shaded regions) of cumulative episode’s reward for the four algorithms in four different environments: (**a**) Cooperation. (**b**) Competition (**c**) Predator–Prey 2v1. (**d**) Predator–Prey 1v2.

Evaluating reliability of the proposed learning frameworks is of significance to verify their applicability in real-world scenarios. A reliable learning procedure should be able to provide consistency in its performance and generate reproducible results over multiple runs of the model [48]. Generally speaking, performance of RL-based solutions, particularly DNN-based approaches, are highly variable because of their dependence on a large number of tunable parameters. Hyperparameters, implementation details, and environmental factors are among these parameters [61]. This can result in unreliability of DNN-based RL algorithms in real-world scenarios compared to the proposed frameworks that are less dependent on parameter selection and fine-tuning. To better illustrate reliability of the proposed frameworks, another experiment is conduced where the initial parameters in each run are generated randomly. More specifically, we have repeated each test 10 times consisting of 1000 learning episodes together with 1000 test episodes. A reliable RL algorithm should be consistent in regenerating performance across different training sessions, i.e., reproducibility feature. As can be seen from Figure 6, for all four test scenarios (i.e., cooperative, competitive, and mixed strategies) DNN-based methods (MADDPG, DDPG, and DQN) have higher variance illustrating their sensitivity to the underlying parameters that can be attributed to reduced reliability. As can be seen from Figure 6, MAK-SR outperforms other approaches in terms of the received awards. In both MAK-SR and MAK-TD algorithms, positive effect of uncertainty usage in the action selection procedure is noticeable. The ability to produce stable performance across different episodes is another aspect for investigating reliability of RL models. Stability of different models can also be compared through Figure 6. It can be seen that the proposed MAK-SR algorithm is more stable than its counterparts as fewer sudden changes occur during different episodes.

With regards to potential future works, on the one hand, the proposed frameworks can be implemented and applied to higher-dimensional MARL environments, e.g., large-scale IoT applications such as indoor localization scenarios in unconstrained environments. One interesting scenario here is to consider a heterogenous network of multiple agents using different tracking/localization algorithms with application to Contact Tracing (CT). Another direction for future research is to focus on optimization of the current SR-based solution. In its current form, the SR weight matrix is approximated by mapping into a one-dimensional vector and applying KF leveraging the KTD framework. For application to higher dimensions, this vectorized approach can result in potential information loss as such more complex approximation techniques should be developed while being mindful of potential computation overhead.

## 7. Conclusions

The paper proposed the MAK-TD framework and its SR-based variant, the MAK-SR framework, as efficient alternatives to DNN-based MARL solutions. The main objective of these developments is to address sample inefficiency, memory problems, and lack of prior information issues of DNN-based MARL techniques. The novelty of the proposed frameworks lies in the integration of Kalman temporal different, multiple-model adaptive estimation, and successor representation for MARL problems. Through such an integration, aforementioned issues related to overfitting and high sensitivity to parameter selection are addressed and changes in the reward model are accommodated. More specifically, by leveraging the KTD framework, SR learning procedure is modeled into a KF problem and RBFs are used to encode the continuous space into feature vectors. For learning localized reward functions, we resort to MMAE to deal with the lack of prior knowledge on the underlying parameters. Additionally, via learning the value function as the inner product of the SR and the weight vector of the reward function, the models can deal with changes in the reward function. Finally, an innovative active learning mechanism is implemented to use the obtained uncertainty of the value function and establish a trade-off between exploration and exploitation. The proposed MAK-TD/SR frameworks are evaluated via several experiments across four different environments, which are implemented through the OpenAI Gym multi-agent RL benchmarks. In these experiments, different number of agents in cooperative, competitive, and mixed (cooperative-competitive) scenarios are utilized. For evaluation purposes, we looked at the average loss, average accumulative reward, the number of steps, and reproducibility/stability aspects of reliability computed over multiple realizations. Based on the results, the proposed MAK-TD/SR frameworks outperformed their counterparts across different evaluation metrics. For example, for the competition scenario, the MAK-SR achieved total average loss of 0.43, while its DNN-based counterparts achieved total average loss of 10,158.18, 10,710.37, and 107.39 for MADDPG, DDPG, and DQN, respectively. Finally, MAK-TD/SR and MAK-TD require much less time and space to find the best policy, while the other three DNN-based approaches can reach such an efficiency with a much higher amount of experience (more than 10,000 experiments) and need much more memory space to save the batches of the information.

## Figures and Tables

**Table 1:** Total loss averaged across all the episodes and for all the four implemented scenarios.

Environment	MAK-SR	MAK-TD	MADDPG	DDPG	DQN
Cooperation	8.93	2.4088	9649.84	10,561.16	10.93
Competition	0.43	4.9301	10,158.18	10,710.37	107.39
Predator–Prey 1v2	0.005	1.9374	6816.34	6884.33	8.21
Predator–Prey 2v1	8.87	1.2421	7390.18	6882.2	10.24

**Table 2:** Total received reward by the agents averaged for all the four implemented scenarios.

Environment	MAK-SR	MAK-TD	MADDPG	DDPG	DQN
Cooperation	−16.0113	−23.0113	−69.28	−66.29	−39.96
Competition	−0.778	−13.358	−63.30	−61.34	−14.49
Predator–Prey 1v2	−0.0916	−13.432	−46.17	−20.53	−23.451
Predator–Prey 2v1	−0.081	−17.0058	−55.69	−49.41	−44.32

**Table 3:** Average steps taken by agents per episode for all the environments based on the implemented platforms.

Environment	MAK-SR	MAK-TD	MADDPG	DDPG	DQN
Cooperation	14.03	12.064	7.377	7.369	15.142
Competition	17.59	17.48	7.36	7.18	11.98
Predator–Prey 1v2	14.78	12.36	6.21	7.69	10.02
Predator–Prey 2v1	9.94	9.773	6.25	7.12	8.46
